# New Amide Derivatives of Quinoxaline 1,4-di-*N*-Oxide with Leishmanicidal and Antiplasmodial Activities

**DOI:** 10.3390/molecules18044718

**Published:** 2013-04-22

**Authors:** Carlos Barea, Adriana Pabón, Silvia Pérez-Silanes, Silvia Galiano, German Gonzalez, Antonio Monge, Eric Deharo, Ignacio Aldana

**Affiliations:** 1 Unidad de Investigación y Desarrollo de Nuevos Medicamentos, Centro de Investigación en Farmacobiología Aplicada (CIFA), Universidad de Navarra, Pamplona 31080, Spain; 2 Grupo Malaria, Facultad de Medicina, Universidad de Antioquia, 050010 Medellín, Colombia; 3 Programa de Biología, Facultad de Ciencias Básicas, Universidad del Atlántico, 080001 Barranquilla, Colombia; 4 Université de Toulouse, UPS, UMR 152 Pharma-DEV, Université Toulouse 3, Faculté des Sciences Pharmaceutiques, F-31062 Toulouse cedex 09, France; 5 Institut de Recherche pour le Développement (IRD), UMR 152 Pharma-DEV, F-31062 Toulouse cedex 09, France

**Keywords:** quinoxaline, 1,4-di-*N*-oxide, leishmanicidal, antiplasmodial

## Abstract

Malaria and leishmaniasis are two of the World’s most important tropical parasitic diseases. Continuing with our efforts to identify new compounds active against malaria and leishmaniasis, twelve new 1,4-di-*N*-oxide quinoxaline derivatives were synthesized and evaluated for their *in vitro* antimalarial and antileishmanial activity against *Plasmodium falciparum* FCR-3 strain, *Leishmania infantum* and *Leishmania amazonensis*. Their toxicity against VERO cells (normal monkey kidney cells) was also assessed. The results obtained indicate that a cyclopentyl derivative had the best antiplasmodial activity (2.9 µM), while a cyclohexyl derivative (2.5 µM) showed the best activity against *L. amazonensis*, and a 3-chloropropyl derivative (0.7 µM) showed the best results against *L. infantum*. All these compounds also have a Cl substituent in the R^7^ position.

## 1. Introduction

Malaria and leishmaniasis are important, social and economical health problems, particularly in the tropical countries. Malaria is a major public health problem today in more than 106 countries and its prevalence is estimated on the order of 216 million clinical cases annually, with a mortality estimated at 266 thousand persons per year; leishmania is responsible for some 2 million clinical cases each year in 88 countries. Most available drugs against malaria and leishmania are costly, highly toxic, require long treatment regimens and are currently losing their effectiveness due to the development of resistance on the part of the respective parasites. Therefore, new effective and affordable antiplasmodial and leishmanicidal agents are urgently needed [1,2].

Quinoxaline derivatives are a class of compounds of great interest within the field of medicinal chemistry because they display a broad range of biological properties such as anticancer [3,4], antimycobacterial [5,6], anti-inflammatory [7], antiviral [8], antiprotozoal [9,10,11,12] and antibacterial activities [13]. The oxidation of both nitrogens of this heterocyclic system, carried out in order to obtain quinoxaline 1,4-di-*N*-oxide derivatives, increases the range of biological properties [14].

In an attempt to intensify the antiparasitic activity of quinoxaline derivatives, our group has synthesized different series that offer promising results; these consisted in the introduction of a carbonitrile group in position 2, which increases the antiparasitic activity, and an amide group in position 3, with the aim of linking together new molecules with interesting activities [15,16]. Continuing with this strategy, we have synthesized and evaluated *in vitro* twelve new amide derivatives of 1,4-di-*N*-oxide quinoxaline against *Plasmodium falciparum* FCR-3 strain (chloroquine-resistant), against *Leishmania infantum*, responsible for visceral forms, and against *Leishmania amazonensis*, responsible for cutaneous expression of the disease.

## 2. Results and Discussion

### 2.1. Chemistry

**Scheme 1:**
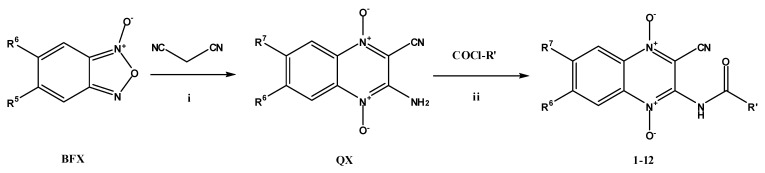
General synthesis of new amide derivatives of quinoxaline 1,4-di-*N*-oxide.

The benzofuroxane starting compounds (BFX, **I**, Scheme 1) have been prepared using previously described methods [17,18]. The 3-amino-1,4-di-*N*-oxide quinoxaline-2-carbonitrile derivatives (QX, **II**) were obtained from the corresponding BFX by the Beirut reaction with malononitrile, using *N,N*-dimethylformamide (DMF) as solvent and triethylamine as catalyst [19].

The method for synthesizing the final compounds consists of reacting 3-amino-2-cyanoquinoxaline 1,4-dioxide derivatives with (purchased) cyclo- and aliphatic-acyl chlorides at room temperature for two hours, using dry tetrahydrofuran as solvent. 

### 2.2. Pharmacology and Structure-Activity Relationship

With regard to the antiplasmodial activity shown in Table 1, halogen groups at R^7^ increase the activity, as shown in previous series of quinoxaline 1,4-di-*N*-oxide derivatives [15]. The cyclopropyl group lowered the antiplasmodial activity almost 100-fold compared to chloroquine (0.2 µM). The cyclopentyl group associated with Cl enhanced the activity (2.9×), but it was still fifteen times less active than chloroquine. When this group was changed for a methyl, acetyl or chloropropyl, the activity decreased to approximately 5 µM. A cyclohexyl group did not enhance the activity (the best one being 7.5 with a Cl substituent). None of the tested compounds showed noticeable toxicity towards VERO cells. 

**Table 1:** Biological characterization of the final compounds. 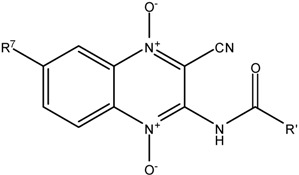

Compound	R^7^	R’	IC_50_ (µM) ^a^	IC_50_ (µM) ^b^	IC_50_ (µM) ^c^	CC_50_ (µM) ^d^	SI ^e^
**1**	H	cyclopropyl	18.3	3.6	-	55.1	15.3
**2**	Cl	cyclopropyl	13.3	3.5	-	144.6	40.8
**3**	CH_3_	cyclopropyl	31	3.5	-	52.8	15
**4**	CH_3_O	cyclopropyl	27.8	3.9	-	145.6	36.7
**5**	Cl	cyclopentyl	2.9	-	14.9	NT	NT
**6**	Cl	cyclohexyl	7.5	2.5	-	249	98
**7**	CH_3_	cyclohexyl	21.6	4.6	-	240.1	52.2
**8**	CH_3_O	cyclohexyl	12.9	3.4	-	238.5	69.1
**9**	H	methyl	6.2	-	16.6	NT	NT
**10**	H	acetyl	5.3	-	11.9	NT	NT
**11**	Cl	acetyl	4.3	-	4	NT	NT
**12**	Cl	3-chloropropyl	5.7	-	0.7	NT	NT
CQ			0.2				
Amph B				0.2	0.15	13	62.1

^a^ IC_50_ against *P. falciparum* FCR-3; ^b^ IC_50_ against axenic amastigotes of *L. infantum*; ^c^ IC_50_ against axenic amastigotes of *L. amazonensis*; ^d^ Cytotoxicity in VERO cells; ^e^ Selectivity Index (SI): CC_50_ drug^d^/IC_50_ drug^b^. NT: Not tested. CQ: chloroquine. Amph B: amphotericin B.

With regard to leishmanicidal activity, also shown in Table 1, some compounds were tested against *L. infantum* and others against *L. amazonensis*. This choice was made according to preliminary testing results. All compounds assayed against *L. infantum* had some activity and most of them presented low toxicity, especially compounds **6** and **8**, which showed better selectivity indexes than amphotericin B. Compound **6** was the most active, but it was ten times less active than amphotericin B. Among the compounds assayed against *Leishmania amazonensis*, compound **12** showed an activity which was only 5 times lower than the activity shown by amphotericin B. Interestingly, the presence of halogen groups at R^7^ and an increase in the length of the aliphatic chain are correlated with increasing anti-malarial and leishmanicidal activity. As *Leishmania* species are known to harbor different sensitivity against leishmanicidal compounds we plan to perform supplementary studies for all the compounds in both models. Additional cytotoxic evaluation must be conducted prior to *in vivo* testing [20].

## 3. Experimental

### 3.1. Chemical Synthesis

#### 3.1.1. General Remarks

All of the synthesized compounds were chemically characterized by thin layer chromatography (TLC), infrared spectroscopy (IR), proton nuclear magnetic resonance (^1^H-NMR) and elemental microanalyses (CHN). Alugram SIL G/UV254 (Layer: 0.2 mm) (Macherey-Nagel GmbH & Co. KG., Düren, Germany) was used for TLC and Silica gel 60 (0.040–0.063 mm, Merck) was used for Flash Column Chromatography. Automated Flash Column Chromatography was developed on an automated Flash Chromatography System CombiFlash^®^ R_f_ (TELEDYNE ISCO, Lincoln, NE, USA) instrument with Silica RediSep^®^ R_f_ columns (average particle size: 35 to 70 microns; average pore size: 60 Å). Purification methods were developed using dichloromethane and methanol to run suitable gradient conditions. The ^1^H-NMR spectra were recorded on a Bruker 400 Ultrashield instrument (400 MHz), using TMS as internal standard and with DMSO-d_6_ as solvent; the chemical shifts are reported in ppm (δ) and coupling constant (*J*) values are given in Hertz (Hz). Signal multiplicities are represented by: s (singlet), bs (broad singlet), d (doublet), t (triplet), dd (doublet of doublets) and m (multiplet). The IR spectra were recorded on a Nicolet Nexus FTIR (Thermo, Madison, WI, USA) in KBr pellets. Elemental microanalyses were obtained on a CHN-900 Elemental Analyzer (Leco, Tres Cantos, Spain) from vacuum-dried samples. The analytical results for C, H and N, were within ±0.5 of the theoretical values. Chemicals were purchased from Panreac Química S.A. (Barcelona, Spain), Sigma-Aldrich Química S.A. (Alcobendas, Spain), Acros Organics (Janssen Pharmaceutical, Geel, Belgium) and Lancaster (Bischheim-Strasbourg, France).

#### 3.1.2. General Procedure for the Synthesis of Quinoxalines **II**

Malononitrile (18.0 mmol) was added to a solution of the appropriate benzofuroxane (**I**, 15.0 mmol) in DMF (10 mL). The mixture was allowed to stand at 0 °C. Triethylamine (1.5 mL) was added dropwise, and the reaction mixture was stirred at room temperature in darkness for 1 day. The resulting precipitate was filtered off and washed by adding diethyl ether, affording the target compound. The obtained red solid was used in the next step without further purification [21]. The yield of this reaction depends on the substituents in positions 5 and 6 in the benzofuroxane. When quinoxalines were prepared from monosubstituted-BFX, the formation of isomeric quinoxalines 1,4-di-*N*-oxide was observed. In most cases, the 7-substituted isomer prevailed over 6-substituted isomer, and when the methoxy substituted quinoxalines were prepared, only the 7-isomer was obtained, as previously described [22,23]. 

#### 3.1.3. General Procedure for the Synthesis of New Amide Derivatives of Quinoxaline 1,4-di-*N*-Oxide

An excess of the corresponding carbonyl chloride (1:1.2) was added to a stirred solution of 3-amino-1,4-di-*N*-oxide quinoxaline-2-carbonitrile derivative (5 mmol) in dry tetrahydrofuran (60 mL). The resulting mixture was stirred at room temperature for 2 h and the solid was collected and purified by column chromatography (dichloromethane/methanol 97:3 or toluene/dioxane 6:4). Finally, the solvents were removed *in vacuo* and the solid precipitated with cold diethyl ether, filtered off in order to obtain a yellow or orange solid [15]. 

*2-Cyano-3-(cyclopropanecarboxamido)quinoxaline 1,4-dioxide* (**1**). Yield 21%; ^1^H-NMR δ ppm: 11.54 (s, 1H, NH); 8.52 (d, 1H, H_8_, *J*_8-7_ = 8.3 Hz); 8.45 (d, 1H, H_5_, *J*_5-6_ = 8.3 Hz); 8.08 (t, 1H, H_7_, *J*_7-8_ = 8.3 Hz, *J*_7-6_ = 8.3 Hz); 7.99 (t, 1H, H_6_, *J*_6-7_ = 8.3 Hz, *J*_6-5_ = 8.3 Hz); 2.26 (m, 1H, CH); 0.98 (d, 2H, CH_2_); 0.92 (d, 2H, CH_2_); IR ν cm^−1^: 3250 (m, NH); 2373 (w, C≡N); 1692 (s, C=O); 1332 (s, N^+^O^−^); Anal. Calc. for C_13_H_10_N_4_O_3_: C: 57.77%; H: 3.70%; N: 20.74%. Found: C: 57.28%; H: 3.82%; N: 20.44%.

*7-Chloro-2-cyano-3-(cyclopropanecarboxamido)quinoxaline 1,4-dioxide* (**2**). Yield 20%; ^1^H-NMR δ ppm: 11.64 (s, 1H, NH); 8.50 (d, 1H, H_8_, *J*_8-6_ = 2.2 Hz); 8.45 (d, 1H, H_5_, *J*_5-6_ = 9.3 Hz); 8.02 (dd, 1H, H_6_, *J*_6-8_ = 2.2 Hz, *J*_6-5_ = 9.3 Hz); 2.27 (m, 1H, CH); 0.99 (d, 2H, CH_2_); 0.93 (d, 2H, CH_2_); IR ν cm^−1^: 3245 (m, NH); 2371 (w, C≡N); 1687 (s, C=O); 1326 (s, N^+^O^−^); Anal. Calc. for C_13_H_9_N_4_O_3_Cl: C: 51.23%; H: 2.95%; N: 18.39%. Found: C: 50.86%; H: 3.28%; N: 18.37%.

*2-Cyano-3-(cyclopropanecarboxamido)-7-methylquinoxaline 1,4-dioxide* (**3**). Yield 23%; ^1^H-NMR δ ppm: 11.49 (s, 1H, NH); 8.39 (d, 1H, H_5_, *J*_5-6_ = 8.6 Hz); 8.32 (d, 1H, H_6_, *J*_6-5_ = 8.6 Hz); 8.26 (s, 1H, H_8_); 2.58 (s, 3H, CH_3_-C7); 2.25 (bs, 1H, CH); 0.97 (bs, 2H, CH_2_); 0.91 (bs, 2H, CH_2_); IR ν cm^−1^: 3247 (m, NH); 2312 (w, C≡N); 1685 (s, C=O); 1328 (s, N^+^O^−^); Anal. Calc. for C_14_H_12_N_4_O_3_: C: 59.15%; H: 4.22%; N: 19.71%. Found: C: 59.09%; H: 4.65%; N: 19.30%.

*2-Cyano-3-(cyclopropanecarboxamido)-7-methoxyquinoxaline 1,4-dioxide* (**4**). Yield 25%; ^1^H-NMR δ ppm: 11.42 (s,1H, NH); 8.43 (d, 1H, H_5_, *J*_5-6_ = 9.4 Hz); 7.74 (d, 1H, H_8_, *J*_8-6_ = 2.7 Hz); 7.69 (dd, 1H, H_6_, *J*_6-8_ = 2.7 Hz, *J*_6-5_ = 9.4 Hz); 4.01 (s, 3H, CH_3_O); 2.51 (bs, 1H, CH); 0.97 (d, 2H, CH_2_); 0.91 (d, 2H, CH_2_); IR ν cm^−1^: 3256 (m, NH); 2370 (w, C≡N); 1691 (s, C=O); 1327 (s, N^+^O^−^); Anal. Calc. for C_14_H_12_N_4_O_4_: C: 56.00%; H: 4.00%; N: 18.66%. Found: C: 55.61%; H: 4.12%; N: 18.19%.

*7-Chloro-2-cyano-3-(cyclopentanecarboxamido)quinoxaline 1,4-dioxide* (**5**). Yield 25%; ^1^H-NMR δ ppm: 11.24 (s, 1H, NH); 8.49 (d, 1H, H_5_ QX, *J*_5-6_ = 9.2 Hz); 8.46 (d, 1H, H_8_ QX, *J*_8-6_ = 2.2 Hz); 8.10 (dd, 1H, H_6_ QX, *J*_6-8_ = 2.2 Hz, *J*_6-5_ = 9.2 Hz); 3.18 (m, 1H, CH); 1.90 (bs, 2H, H_2_+H_5_ eq. cyclo); 1.81 (bs, 2H, H_2_+H_5_ ax. cyclo); 1.68 (bs, 2H, H_3_+H_4_ eq. cyclo); 1.60 (bs, 2H, H_3_+H_4_ ax. cyclo); IR ν cm^−1^: 3307 (m, NH); 2315 (w, C≡N); 1701 (s, C=O); 1327 (s, N^+^O^−^); Anal. Calc. for C_15_H_13_N_4_O_3_Cl: C: 54.13%; H: 3.90%; N: 16.84%. Found: C: 53.90%; H: 3.77%; N: 17.10%.

*7-Chloro-2-cyano-3-(cyclohexanecarboxamido)quinoxaline 1,4-dioxide* (**6**). Yield 17%; ^1^H-NMR δ ppm: 11.19 (s, 1H, NH); 8.48 (d, 1H, H_5_ QX, *J*_5-6_ = 9.2 Hz); 8.46 (d, 1H, H_8_ QX, *J*_8-6_ = 2.2 Hz); 8.10 (dd, 1H, H_6_ QX, *J*_6-8_ = 2.2 Hz, *J*_6-5_ = 9.2 Hz); 2.72 (m, 1H, CH); 1.86 (d, 2H, H_2_+H_6_ eq. cyclo); 1.78 (d, 2H, H_3_+H_5_ eq. cyclo); 1.65 (d, 2H, H_2_+H_6_ ax. cyclo); 1.43 (m, 2H, H_3_+H_5_ ax. cyclo); 1.25 (d, 2H, CH_2_^4^ cyclo); IR ν cm^−1^: 3286 (m, NH); 2236 (w, C≡N); 1696 (s, C=O); 1327 (s, N^+^O^−^); Anal. Calc. for C_16_H_15_N_4_O_3_Cl: C: 55.41%; H: 4.32%; N: 16.16%. Found: C: 54.95%; H: 4.59%; N: 16.00%.

*2-Cyano-3-(cyclohexanecarboxamido)-7-methylquinoxaline 1,4-dioxide* (**7**). Yield 11%; ^1^H-NMR δ ppm: 11.07 (s, 1H, NH); 8.03 (d, 1H, H_5_ QX, *J*_5-6_ = 8.7 Hz); 7.32 (s, 1H, H_8_ QX); 7.27 (dd, 1H, H_6_ QX, *J*_6-8_ = 1.3 Hz, *J*_6-5_ = 8.7 Hz); 2.71 (m, 1H, CH); 2.51 (s, 3H, CH_3_-C7 QX); 1.87 (d, 2H, H_2_+H_6_ eq. cyclo); 1.78 (d, 2H, H_3_+H_5_ eq. cyclo); 1.65 (d, 2H, H_2_+H_6_ ax. cyclo); 1.44 (d, 2H, H_3_+H_5_ ax. cyclo); 1.27 (dd, 2H, CH_2_^4^ cyclo); IR ν cm^−1^: 3248 (m, NH); 2374 (w, C≡N); 1691 (s, C=O); 1327 (s, N^+^O^−^); Anal. Calc. for C_17_H_18_N_4_O_3_: C: 62.57%; H: 5.52%; N: 17.17%. Found: C: 62.09%; H: 5.43%; N: 16.76%.

*2-Cyano-3-(cyclohexanecarboxamido)-7-methoxyquinoxaline 1,4-dioxide* (**8**). Yield 30%; ^1^H-NMR δ ppm: 11.01 (s, 1H, NH); 8.41 (d, 1H, H_5_ QX, *J*_5-6_ = 9.4 Hz); 7.74 (d, 1H, H_8_ QX, *J*_8-6_ = 2.7 Hz); 7.69 (dd, 1H, H_6_ QX, *J*_6-8_ = 2.7 Hz, *J*_6-5_ = 9.4 Hz); 4.01 (s, 3H, CH_3_O); 2.69 (m, 1H, CH); 1.87 (d, 2H, H_2_+H_6_ eq. cyclo); 1.77 (d, 2H, H_3_+H_5_ eq. cyclo); 1.65 (d, 1H, H_2_+H_6_ ax. cyclo); 1.44 (m, 2H, H_3_+H_5_ ax. cyclo); 1.26 (d, 2H, CH_2_^4^ cyclo); IR ν cm^−1^: 3245 (m, NH); 2373 (w, C≡N); 1691 (s, C=O); 1327 (s, N^+^O^−^); Anal. Calc. for C_17_H_18_N_4_O_4_: C: 59.64%; H: 5.26%; N: 16.37%. Found: C: 59.20%; H: 5.34%; N: 16.33%.

*3-Acetamido-2-cyanoquinoxaline 1,4-dioxide* (**9**). Yield 15%; ^1^H-NMR δ ppm: 11.29 (s, 1H, NH); 8.50 (d, 1H, H_5_, *J*_5-6_ = 8.5 Hz); 8.45 (d, 1H, H_8_, *J*_8-7_ = 8.5 Hz); 8.08 (t, 1H, H_6_, *J*_6-7_ = 7.6 Hz); 7.99 (t, 1H, H_7_); 2.27 (s, 3H, CH_3_); IR ν cm^−1^: 3256 (m, NH); 2374 (w, C≡N); 1524 (s, C=O); 1331 (s, N^+^O^−^); Anal. Calc. for C_11_H_8_N_4_O_3_: C: 54.09%; H: 3.27%; N: 22.95%. Found: C: 53.74%; H: 3.02%; N: 23.43%.

*2-Cyano-3-propionamidoquinoxaline 1,4-dioxide* (**10**). Yield 15%; ^1^H-NMR δ ppm: 11.26 (s, 1H, NH); 8.51 (d, 1H, H_5_, *J*_5-6_ = 8.6 Hz); 8.46 (d, 1H, H_8_, *J*_8-7_ = 8.6 Hz); 8.08 (t, 1H, H_7_); 8.01 (dd, 1H, H_6_, *J*_6-5_= 8.6 Hz); 2.58 (d, 2H, CH_2_, *J*_CH2-CH3_ = 7.4 Hz); 1.13 (t, 3H, CH_3_, *J*_CH3-CH2_ = 7.4 Hz); IR ν cm^−1^: 3250 (m, NH); 2236 (w, C≡N); 1524 (s, C=O); 1333 (s, N^+^O^−^); Anal. Calc. for C_12_H_10_N_4_O_3_: C: 55.81%; H: 3.87%; N: 21.70%. Found: C: 56.05%; H: 3.66%; N: 22.10%.

*7-Chloro-2-cyano-3-propionamidoquinoxaline 1,4-dioxide* (**11**). Yield 5%; ^1^H-NMR δ ppm: 11.28 (s, 1H, NH); 8.49 (d, 1H, H_5_, *J*_5-6_ = 8.6 Hz); 8.46 (d, 1H, H_8_); 8.09 (dd, 1H, H_6_, *J*_6-8_ = 2.4 Hz, *J*_6-5_ = 8.6 Hz); 2.58 (d, 2H, CH_2_, *J*_CH2-CH3_ = 7.2 Hz); 1.13 (t, 3H, CH_3_, *J*_CH3-CH2_ = 7.2 Hz); IR ν cm^−1^: 3254 (m, NH); 2366 (w, C≡N); 1517 (s, C=O); 1321 (s, N^+^O^−^); Anal. Calc. for C_12_H_9_N_4_O_3_Cl: C: 49.23%; H: 3.07%; N: 19.14%. Found: C: 49.18%; H: 2.84%; N: 19.18%.

*7-Chloro-3-(4-chlorobutanamido)-2-cyanoquinoxaline 1,4-dioxide* (**12**). Yield 5%; ^1^H-NMR δ ppm: 11.44 (s, 1H, NH); 8.50 (d, 1H, H_5_, *J*_5-6_ = 9.1 Hz); 8.47 (d, 1H, H_8_, *J*_8-6_ = 2.2 Hz); 8.11 (dd, 1H, H_6_, *J*_6-8_ = 2.2 Hz, *J*_6-5_ = 9.1 Hz); 3.73 (t, 2H, CH_2_^3^); 2.75 (t, 2H, CH_2_^2^, *J*_2-3_ = 7.0 Hz, *J*_2-1_ = 7.0 Hz); 2.08 (t, 2H, CH_2_^1^); IR ν cm^−1^: 3256 (m, NH); 2373 (w, C≡N); 1517 (s, C=O); 1324 (s, N^+^O^−^); Anal. Calc. for C_13_H_10_N_4_O_3_Cl_2_: C: 45.74%; H: 2.93%; N: 16.42%. Found: C: 45.39%; H: 2.83%; N: 16.48%.

### 3.2. Pharmacology

#### 3.2.1. *In Vitro* Antiplasmodial Drug Assay

Chloroquine-resistant FCR-3 strain of *P. falciparum* was cultivated at 37 °C in a 5% CO_2_ environment in glucose-enriched RPMI 1640 medium supplemented with gentamicin 0.1 mg/mL and 10% heat-inactivated A^+^ human serum, as previously described [24]. The drugs, dissolved in dimethylsulfoxide (DMSO), were added at final concentrations ranging from 250 to 0.1 µM. The final DMSO concentration was never greater than 0.1%. *In vitro* antimalarial activity was measured using the [3H]-hypoxanthine (MP Biomedicals, Santa Ana, CA, USA) incorporation assay [25]. Briefly, 250 µL of total culture medium with the diluted drug and the suspension of human red blood cells in medium (A^+^ group, 5% hematocrit) with 1% parasitaemia were placed into the wells of 96-well microtiter plates. On the third day of the test, radioactivity was assessed. All experiments were performed in triplicate. Results were expressed as the concentration resulting in 50% inhibition (IC_50_), which was calculated by a nonlinear regression logistic dose response model; the mean IC_50_ values and standard deviation for each compound were calculated.

#### 3.2.2. *In Vitro* Cytotoxicity

Toxicity was determined using Vero cells (normal monkey kidney cells) cultured under the same conditions as *P. falciparum*, except for the replacement of 5% human serum with 10% fetal calf serum. After the addition of compounds at increasing concentrations, cell growth was measured by [^3^H]-hypoxanthine incorporation after a 48-h incubation period and then compared with a control sample [26].

#### 3.2.3. *In Vitro* Antileishmanial Drug Assay

Leishmanicidal activity was determined on axenic cultures of *L. infantum* and *amazonensis* amastigotes. In order to estimate the 50% inhibitory concentration (IC_50_) of the drugs, the 3-(4,5-dimethylthiazol-2-yl)-2,5-diphenyltetrazolium bromide (MTT) micromethod was used as previously described [27]. Briefly, *Leishmania* strain was maintained in promastigote stage in a biphasic medium (blood agar with 0.89% NaCl, pH 7.4) at 24 °C, with sub-passage every 3–4 days. Promastigotes (5 × 10^6^ parasites) were then transferred to M199 medium supplemented with 10% fetal bovine serum, pH 7.4. After 4 days, exponential phase promastigotes were centrifuged for 10 min at 1,500 g and 4 °C. The supernatant was discarded and replaced by fresh M199 medium supplemented with 20% FBS, pH 5.5. Axenic amastigote transformation was then induced by increasing the temperature to 34 °C. Drugs were then tested at increasing concentrations. 

## 4. Conclusions

Compounds **5**, **6** and **12** were the most active against *Plasmodium falciparum*, *Leishmania infantum* and *L. amazonensis*, respectively. The presence of a halogenous atom at position 7 and the increase of the aliphatic chain length increase the level of activity. Therefore, these compounds have been selected as lead compounds in the future design of new compounds against *Plasmodium and Leishmania*.
